# Intention to Transfer and Transfer Following eLearning in Spain

**DOI:** 10.1007/s12186-022-09292-w

**Published:** 2022-06-13

**Authors:** Carla Quesada-Pallarès, Aitana González-Ortiz-de-Zárate, Pilar Pineda-Herrero, Eduardo Cascallar

**Affiliations:** 1 Applied Pedagogy Department, Autonomous University of Barcelona, Barcelona, Catalonia Spain; 2 Present Address: Serra Hunter Fellow, Barcelona, Spain; 3 Present Address: Work Sciences Department, Universidad a Distancia de Madrid, Madrid, Spain; 4 Present Address: Educational Theories and Social Pedagogy Department, Autonomous University of Barcelona, Barcelona, Catalonia Spain; 5 Present Address: Faculty of Psychology and Educational Sciences, KU Leuven University, Leuven, Belgium

**Keywords:** Learning transfer, Training transfer, Intention to transfer, Motivation, Cluster analysis

## Abstract

Understanding vocational learning and transfer is vital to European citizens. We need to understand how transfer works, which factors influence it, and how these factors affect employee behaviour. Research in online training specific to Southern Europe is needed to move the field forward. The Unified Model of Motivation for Training Transfer (MTT) was proposed to understand behaviour change after training. It conceives three phases: (1) forming transfer intentions, (2) actualizing implementation intentions for transfer, and (3) strengthening transfer commitment. We analysed initial transfer intention and transfer following online training in three Spanish organisations. We used an ex post facto prospective design with one group (*n* = 204). We applied the online version of the Initial Transfer Intention questionnaire (ITI) three days before the training, and the Transfer Questionnaire (TrQ) three to four months after the training. Training consisted of 22 online courses offered by the three participating organisations. A cluster analysis and post hoc analysis were performed. We identified three groups (k = 3), indicating that there were significant differences in the means between employees with low and high intention to transfer. Results showed a greater difference in the factor profile between participants with LowPT and HighPT. We identified common characteristics among people with low levels of transfer; this information can help understand what type of employee will transfer less and provide cues on how to prevent this from happening in future training activities. Limitations and recommendations for research and practice are discussed.

The European Commission states that a focus on adult learning is vital for Europe to overcome economic challenges and respond to the demand for new skills and sustained productivity (European Commission, 2021). Working individuals must rely on continuous professional development to remain competitive on the labour market.

For training to be effective, it should be transferred to the job. Learning transfer or training transfer refers to the employee’s level of application of what they learnt in training into the workplace (Ford et al., 2018); it explores employees’ behavioural change due to learning acquired in training activities (Reinhold et al., 2018).

Although research in transfer started more than 30 years ago, we still need to understand how transfer works, which factors influence it, and how these factors affect employee’s behaviours (e. g., Blume et al., 2019; Huang et al., 2015; Massenberg et al., 2017). The study of transfer and the factors that influence transfer requires the application of longitudinal designs; however, such research designs have proven to be scarce in the transfer field (Schoeb et al., 2020).

In the more than 30 years of transfer research, the online teaching modality has been progressively gaining strength. It grew 900% in the past 20 years (Global Industry Analysts, 2020), becoming more and more frequent in organisations. In addition, the COVID-19 pandemic cut for months face-to-face interactions and temporarily positioned online delivery as the only possibility to facilitate training (Soni, 2020).

Additionally, it has been estimated that online training can cut energy consumption by 90% (Global Industry Analysts, 2020), positioning this mode of instruction as the sustainable alternative. Knowing that organisations are aligning their strategies with the UN’s sustainable development goals (Rosati & Faria, 2019), aiming at reducing consumption and resources, online training seems to have a projected well-established position in the future of organisations. In this scenario, research in transfer and transfer factors in online environments is a key necessity that can contribute to move the field forward.

In the HRD field in general, and in the transfer field in particular, USA and Western European perspectives have dominated literature (Garavan et al., 2016). Carrying out research in Southern Europe could add evidence from different cultural backgrounds, which could help to build a more globalised perspective. Within Southern Europe, Spain is one of the main countries and a highly active member of the European project (European Union, 2022). Being Spanish the second language in the number of native speakers around the world, with the cultural connections that language implies, makes the study of transfer in Spain an interesting opportunity.

## eLearning

Online learning, also named eLearning, digital learning and virtual learning is the learning that occurs through technology and the internet (Ozuorcun & Tabak, 2012). It is the most common instructional method in distance education (Traxler, 2018). During the past decade, eLearning has been developed in different formats, such as synchronous and asynchronous training, including simulations (Hallinger et al., 2020), active strategies (Monteiro et al., 2020) and gamification (Huang et al., 2010; Klaudia & Bastiaens, 2020). The Small Private Online Courses (SPOC) and the Massive Open Online Courses (MOOC) have appeared (Aparicio et al., 2014; Lan & Hew, 2020). New generations demand flexibility and expect at least some of the work and training to be delivered online (Bennett & McWhorter, 2021). Additionally, the COVID-19 pandemic positioned eLearning as the main alternative to face-to-face formats (Naciri et al., 2020).

Despite these advancements, eLearning effects in transfer should be consistently studied (Sinclair et al., 2016), especially at the level of behaviours and organisations (Salter et al., 2014).

## Learning Transfer

Learning transfer and training transfer have been studied for more than 30 years (e. g., Baldwin & Ford, 1988; Baldwin et al., 2017). At this point, researchers appear to have obtained some consensus via empirical research and meta-analysis, including the assumption that there are individual differences involved (Ford et al., 2018).

Based on the existence of individual differences, researchers have stressed the need for applying a trainee/centred focus when studying learning transfer (Massenberg et al., 2017; Poell, 2017). This trainee/centred focus might include the study of motivation (Gegenfurtner, 2011; Reinhold et al., 2018), initial intention to transfer, and subjective norms (Cheng et al., 2015; Testers et al., 2019).

It has been theorised that intention to transfer might influence the initial attempts to utilise transfer, that is, the first transfer experience (Blume et al., 2019). In addition, it appears to be a critical determinant of training effectiveness (Al-Swidi & Al Yahya, 2017), and it has been found mediating the relationship between the antecedents and transfer behaviour (Cheng et al., 2015).

## Background, The Unified Model of Motivation for Training Transfer (MTT)

**Fig. 1:**
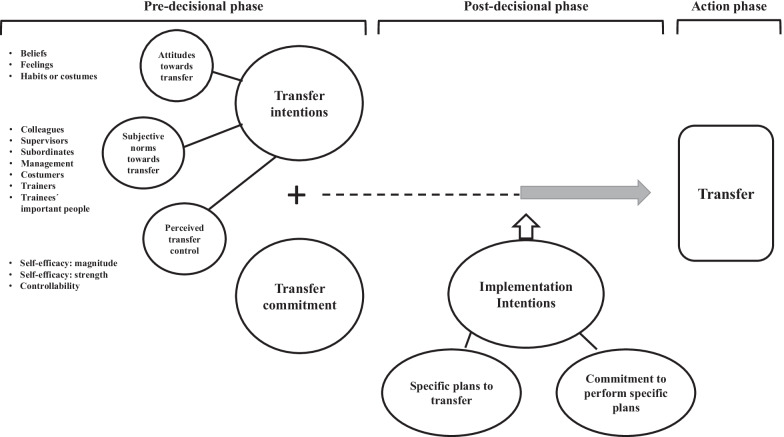
Unified Model of Motivation for Training Transfer (MTT) (Quesada-Pallarès & Gegenfurtner, 2015, p.115)

To better understand intention to transfer and transfer, the Unified Model of Motivation for Training Transfer (MTT) was proposed (Quesada-Pallarès & Gegenfurtner, 2015). The MTT is a model for understanding behaviour change after training. The model integrates elements from classic motivation theories and conceives three phases: (1) forming transfer intentions influenced by attitudes, norms, and perceived transfer control, (2) actualizing implementation intentions for transfer, and (3) strengthening transfer commitment. Figure 1 shows the MTT model.

### Forming Transfer Intentions

Transfer intentions are influenced by attitudes, norms, and perceived transfer control.

*Attitudes towards transfer* are trainees’ attitudes to transfer, which are determined by cognitive, affective, and behavioural elements (Quesada-Pallarès & Gegenfurtner, 2015).

*Subjective norms towards transfer* refers to the social pressure felt by trainees when transferring (Wallace et al., 2005). It includes seven sources of normative influence: colleagues and peers, supervisors, subordinates, management, clients (customers or consumers), the trainer, and other relevant people for the employee (Quesada-Pallarès & Gegenfurtner, 2015).

*Perceived behavioural control* includes trainees’ perceived difficulty in transferring, in overcoming obstacles or barriers during transfer, and trainees’ control when steering transfer. Research has found robust results when using self-efficacy to predict transfer among different training contexts (Gegenfurtner et al., 2014).

### Actualizing implementation intentions for transfer

*Transfer commitment* measures trainees’ commitment to transfer intentions, a necessary antecedent to achieve transfer (Gegenfurtner, 2013; Pineda-Herrero et al., 2014). It explores the importance given by the trainee to the transfer.

### Strengthening Transfer Commitment

The model differentiated two types of behavioural intentions: intention to transfer in a pre-decisional or deliberative phase and implementation intention in a post-decisional or implemental phase. The second phase preceded the action and involved how trainees prepare and implement the action plans (Heckhausen & Gollwitzer, 1987). Here, the *commitment to perform specific plans* plays a key role by establishing a link between the trainee and the implementation of the necessary steps to ensure the action plans are executed (Rise et al., 2003).

This phase aims at achieving the intended results. It covers the actions carried out by trainees focused on transferring.

To measure intention to transfer in the MTT, the Initial Transfer Intention (ITI) questionnaire was developed in Spain (Quesada-Pallarès, 2014).

Although different studies have analysed intention to transfer (e.g., Al-Swidi & Al Yahya, 2017; Testers et al., 2019), no identified study has examined the relation between intention to transfer and transfer following eLearning in a Spanish sample.

## Purpose

This study focused on two research questions: (RQ1) what are the levels of transfer in Spanish employees following online training? It refers to the perceived transfer level that a participant achieved, such a low transfer level (e.g. they transferred not much of the learning acquired in training), medium transfer level or high transfer level (e.g. they transferred a lot of the learning acquired in training). And (RQ2) what is the relationship between the initial transfer intention factors and the levels of transfer in Spain when using cluster analysis technique? It refers to the output of applying the cluster analysis technique to identify groups of people with low, medium or high predisposition to transfer based on their scores on the initial transfer intention factors. Because the relation between transfer intention factors and transfer might vary based on the transfer level, we aimed at (1) categorising employees according to their transfer level, and (2) analysing the relation between the initial transfer intention factors and the different transfer levels.

## Methods

In this section, we describe the procedures used, the sample, the training, the instruments, and data analysis.

### Procedure

We used an ex post facto prospective design with one group (Kumatongo & Muzata, 2021) with no manipulation from the researcher. The ITI was administered online three days before the training (t1), and the Transfer Questionnaire (TrQ from now on) three-to-four months after the training (t2).

The research complied with the Regulation (EU) 2016/679 of the European Parliament and of the Council of 27 April 2016 on the protection of natural persons regarding the processing of personal data and on the free movement of such data and repealing Directive 95/46/EC (General Data Protection Regulation). Participants gave their informed consent before completing the instruments and were free to stop answering at any point without completing the surveys.

### Sample

Because we aimed at assessing transfer in Spain, we purposely reached for three large Spanish organisations. Organisations were based in different points of the country: two public organisations based in Middle and Southern Spain, and a private enterprise based in multiple locations across the country. The research team sourced the organisations by establishing direct contact with their human resource development professionals. A non-probabilistic sampling approach (Castro-Martín et al., 2020) was used to maximise the number of participants.

Participants were employees of the mentioned organisations who had attended one of the training courses (*n* = 943). T1 got 430 responses (response rate = 46%); t2 got 282 responses (response rate = 66%).

Because giving personal details was optional to the participants, 78 respondents did not provide information that allowed the longitudinal match between t1 and t2. Hence, the final sample comprised 204 trainees who answered both questionnaires (22% of the initial sample). Based on the estimation of the average response rate for surveys in organisational studies (response rate = 36%) (Baruch, & Holtom, 2008), based on the estimation of an 11% decrease of online surveys than other survey modes (Fan & Yan, 2010), and knowing that the longitudinal design applied tended to increase participant mortality (Gonzalez-Ortiz-de-Zarate & Quesada-Pallarès, 2021), we considered the response rate adequate. We analysed the profile of the participants who dropped-out in the data analysis section.

Respondent information is shown in Table 1.

**Table 1:** Sample Information

	%
Public administration in Middle Spain	60
Public administration in Southern Spain	18
Private enterprise	22
Women	67
Men	33
Technicians	41
Qualified employees	22
Managers	18
Directors	9
Low-qualified employees	4
Postgraduate degrees	8
Bachelor’s degree	78
Lower merits	14

*n* = 202, after screening

Ages ranged from 32- to 59-year-old, with 61% ranging from 40- to 52-year-old. The average age was 46-year-old (SD = 6.60).

### Training

Training consisted of 22 online courses offered by the three organisations. The organisations classified the courses into three main categories: hard skills (53%), technological skills (31%), and soft skills (16%). Examples of the courses were Implementing Quality Management Systems, Web 2.0, and Effective Thinking. There was an average of 43 students per program (SD = 18.09).

### Instruments

Data was gathered through two online questionnaires using the MTT instruments applied as self-reports: the ITI and the TrQ.

#### Initial Transfer Intention questionnaire (ITI)

The ITI (Quesada-Pallarès, 2014) was developed based on the motivational variables of the MTT that could play an important role in trainees’ intention to transfer. It consists of 96 items and three subscales, containing a total of 14 factors. The three subscales are: (1) initial intention to transfer (12 items, one factor), (2) subjective norms (16 items, four factors), and (3) the rest of transfer intention factors (68 items, nine factors). Answers are given through a 5-point Likert-type scale (1: *not agree or never*, 5: *completely agree or always*).

The subscales were validated in a sample of 667 participants through EFA and CFA following standard procedures (Quesada-Pallarès, 2014). Table 2 shows ITI’s composition and definitions.

**Table 2:** ITI Composition and Definitions

Factor	Definition and item example	Number of items	α^a^	VE
1. Intention to transfer (1)	The degree to which trainees are willing to transfer learning, or how much effort they are willing to make to accomplish this transfer E.g., I want to apply in my work what I learned in training in the next months	12	.97	76%
2. Boss’s desire to transfer (2)	Trainees’ perception about what their boss believes is in relation to the application of what they learned in training to their workplace E.g., My boss wants me to apply what I learned in training to the workplace	3	.97	33%
3. Clients’ desire to transfer (2)	Trainees’ perception about what their clients or customers believe is in relation to applying what they learned in training to their workplace E.g., My clients want me to apply what I learned in training to the workplace’	3	.96	9%
4. Subordinates’ desire to transfer (2)	Trainees’ perception about what their subordinates believe is in relation to applying what they learned in training to their workplace E.g., My subordinates want me to apply what I learned in training to the workplace	3	.93	10%
5. Pressure to transfer (2)	Trainees’ perception about what others believe is in relation to applying what they learned in training to their workplace E.g., I feel pressured by my work colleagues to apply what I learned in training to the workplace	7	.85	19%
6. Overcoming indeterminant obstacles during transfer (3)	The confidence the trainees have when they apply the learnings to their workplace, despite any type of obstacles they may encounter in the process E.g., I believe that I will solve the problems that appear when I apply what I learned in training to the workplace	4	.93	4%
7. Decision-making in the transfer process (3)	Trainees' freedom in deciding whether to apply what they learned in training to their workplace E.g., It's up to me to decide whether or not I apply what I learned in training to the workplace	3	.95	9%
8. Negative feelings towards transfer (3)	Negative feelings that trainees have when they apply the learnings to their workplace E.g., I feel insecure when I apply what I learned in training to the workplace	11	.93	8%
9. Overcoming work environment obstacles during transfer (3)	The confidence trainees have when they apply the learnings to their workplace, despite any work environment obstacles they may encounter in the process E.g., I believe that I will be able to apply what I learned in training to the workplace, even if my work environment doesn’t help	5	.91	5%
10. Habits in the transfer process (3)	Trainees’ usual behaviours when they apply the learnings to their workplace E.g., Before applying what I learned in training to the workplace, I think about how I'm going to do it	11	.93	4%
11. Positive feelings towards transfer (3)	Positive feelings that trainees have when they apply the learnings to their workplace E.g., I feel satisfied when I apply what I learned in training to the workplace	8	.92	4%
12. Beliefs about transfer (3)	Opinions and convictions that trainees have when they apply the learnings to their workplace E.g., I think that the training´s purpose is to apply what I learned during the training to the workplace	17	.95	22%
13. Ability to control transfer (3)	Trainees' have freedom in deciding which way they apply the learnings to their workplace E.g., I can´t decide when to apply what I learned in training to the workplace	5	.92	3%
14. Transfer commitment (3)	The extent to which trainees believe applying what they learned in training to their workplace is a priority E.g., I’m willing to exert effort beyond the usual to apply what I learned in training to the workplace	4	.87	2%

^a^Cronbach’s alpha based on standardised items; VE means Variance Explained; (1) this factor was analysed separately during the exploratory factor analysis because it can be considered a dependent or a mediating variable; (2) these factors were analysed separately during the exploratory factor analysis because of their composition, obtaining a total VE of 70%; (3) these remaining factors were analysed altogether in the exploratory factor analysis, obtaining a total VE of 61%.

#### Transfer Questionnaire (TrQ)

The TrQ measures transfer from the participants’ perspective with the goal of identifying the degree to which a trainee applied knowledge, skills and attitudes learned in training to the workplace. It consists of six items and a single factor. Answers are given through a 5-point Likert-type scale (1: *not agree*; 5: *completely agree*). It was validated through EFA (*n* = 282) and CFA (*n* = 70), showing a high internal consistency (α = 0.92) and following standard procedures (Quesada-Pallarès, 2014). An example of an item is *Due to the training, I have modified my job performance*. An early version of the TrQ was developed by Quesada-Pallarès et al. (2015).

### Data Analysis

SPSS was used for the analysis. Exploratory analyses were performed to ensure sample’s normality and detect outliers. Descriptive statistics and correlation matrix are shown in Table 3.

**Table 3:** Correlation Matrix Among the MTT Factors

	F1	F2	F3	F4	F5	F6	F7	F8	F9	F10	F11	F12	F13	F14	Transfer
F1	3.27 (0.66)														
F2	0.23	3.12 (1.03)													
F3	-0.17	-0.19	1.51 (0.51)												
F4	0.40^**^	0.30^*^	0.02	2.80 (0.71)											
F5	0.35^*^	0.10	0.10	0.25	3.7(0.62)										
F6	0.47^**^	0.03	-0.15	0.14	0.34^*^	3.9 (0.69)									
F7	0.57^**^	0.21	-0.13	0.25	0.37^**^	0.61^**^	3.80 (0.61)								
F8	0.23	0.52^**^	-0.25	0.30^*^	0.32^*^	0.23	0.35^*^	3.87 (0.81)							
F9	0.42^**^	0.19	-0.16	0.13	0.32^*^	0.49^**^	0.41^**^	0.24	3.19 (0.76)						
F10	0.04	0.12	-0.20	0.06	0.18	0.04	0.34^*^	0.19	0.05	3.17 (1.00)					
F11	0.11	0.01	0.03	0.08	0.32^*^	0.12	0.30^*^	0.25	0.20	0.20	2.83 (0.95)				
F12	0.12	-0.11	-0.23	-0.15	-0.02	0.18	0.24	0.01	0.01	0.34^*^	0.49^**^	3.13 (0.75)			
F13	-0.15	-0.33^*^	0.49^**^	-0.05	0.09	-0.26	-0.04	-0.23	-0.22	0.23	0.18	0.12	1.58 (0.52)		
F14	0.41^**^	0.30^*^	-0.35^*^	0.08	0.12	0.60^**^	0.50^**^	0.32^*^	0.63^**^	0.11	0.19	0.07	-0.38^**^	3.8 (0.77)	
Transfer	0.14	-0.19	-0.06	-0.01	0.08	0.38^**^	0.15	-0.01	0.37^**^	-0.11	0.28^*^	0.03	-0.03	0.27	2.81 (0.84)

*n* = 204, after screening. Values in the diagonal refer to the mean and standard deviation (in parenthesis) of each factor; **Correlation is significant at the .01 level (2-tailed); * Correlation is significant at the .05 level (2-tailed); F1: Overcoming indeterminate obstacles during transfer; F2: Decision-making in the transfer process; F3: Negative feelings towards transfer; F4: Overcoming work environment obstacles during transfer; F5: Habits in the transfer process; F6: Positive feelings towards transfer; F7: Beliefs about transfer; F8: Ability to control transfer; F9: Transfer commitment; F10: Boss’s desire to transfer; F11: Clients’ desire to transfer; F12: Subordinates’ desire to transfer; F13: Pressure to transfer; F14: Intention to transfer

Because we wanted to categorise participants according to their transfer level, we applied a cluster analysis technique, which allowed us to group participants in clusters based on their level of transfer.

Given the goal of this study, we chose a non-hierarchical procedure, which partitions the data in non-overlapping sets without hierarchical relationships between them. The K-means approach was selected due to its application in cases where it can be suspected that different participants might perform the task differently, but where we have no external indicator of the subsets except for performance on the task (Farrell & Lewandowsky, 2018). Given a fixed number (k) of clusters, each observation was assigned to one of the clusters, so that the means across clusters (for all variables being considered), were as different from each other as possible. The difference between observations was measured in terms of one of several distance measures (Euclidean distances). To validate the number of clusters we used *distance* in cluster analysis. We carried out a cross-validation to a range of the numbers of clusters and observed the resulting average distance of the observations (in the cross-validation subsample) from the corresponding cluster centres. Because we chose the cluster structure with the best fit to the data, as demonstrated by the smallest average distance from their centres, the solution of 3 clusters was adopted: 3 levels of transfer.

Following the identification of clusters and the employees who belonged to each of the clusters, we conducted post-hoc analysis, such as contingency tables and inferential analysis. For the contingency tables, we used the chi-square test to analyse the degree in which the three clusters (predisposition to transfer levels) were associated to the transfer levels. Regarding the inferential analysis, we used the One-way ANOVA test (with Bonferroni correction) to explore if the fourteen ITI factors were behaving significantly different among the three clusters.

Finally, we explored how participants who did not respond to the TrQ (*n* = 148) behave on the initial transfer intention factors (these participants were not included in the longitudinal study). We applied a t-test by comparing the fourteen ITI factors between the drop-out cohort and the completed cohort. Even though participants from the drop-out cohort tend to show lower scores than the completed cohort, only three factors showed significant differences. The drop-out cohort informed of a significantly lower intention to transfer (M = 3.61, SD = 0.76) than those from the completed cohort (M = 3.78, SD = 0.77), t(374) = -2.123, *p* = .034. Similarly, the drop-out cohort felt having significantly less habits in the habits process (M = 3.61, SD = 0.66) than those from the completed cohort (M = 3.74, SD = 0.62), t(404) = -1.982, *p* = .048. Nonetheless, it was the drop-out cohort participants who thought their boss had significantly higher desire to transfer (M = 3.37, SD = 0.88), compared to the those from the completed cohort (M = 3.17, SD = 1.00), t(373) = 2.108, *p* = .036. Therefore, the drop-out cohort felt a higher pressure from their bosses to transfer and at the same time, felt less prepared to deal with transfer and showed less intention to transfer. We should remember that employees from the drop-put cohort might (or might not) have been part of the training.

## Results

Results are provided in this section: categorization of trainees by their transfer level and clusters’ post-hoc analysis.

### Categorising the Trainees by Their Learning Transfer Level

We sought the number of clusters that best described the common motivational characteristics of trainees on their transfer through the *K*-mean method. The 14 factors were introduced in the analysis, establishing the number of clusters at two (*k* = 2), three (*k* = 3) and four (*k* = 4), in separate analyses. The solution with 3 clusters was chosen considering results related to number of iterations, centroids distances, factors involved in clusters’ establishment, and the M^2^error of the ANOVA: the two clusters solution had a larger error, whereas the error did not change between the three and four cluster solutions. Thus, we report the results of the *k* = 3 analysis.

After eight iterations, the centroids of the three clusters did not vary substantially. Cluster 1 and 3 showed the greatest differences between the centroids of the final clusters (3.53), while cluster 2 had smaller distances to clusters 1 (2.09) and 3 (2.37). *Boss’ desire to transfe*r (0.73) and *clients’ desire to transfe*r (0.60) had less in common with the clusters established in the analysis, as shown in Table 4.

**Table 4:** ANOVA’s Values of Cluster Analysis

Factor	Cluster		Error		F	Sig
	Root mean	Degrees of freedom	Root mean	Degrees of freedom		
Overcoming indeterminate obstacles during transfer	14.30	2	0.29	201	48.72	.000
Decision-making in the transfer process	48.91	2	0.59	201	82.833	.000
Negative feelings towards transfer	1.46	2	0.25	201	5.76	.004
Overcoming work environment obstacles during transfer	7.48	2	0.43	201	17.41	.000
Habits in the transfer process	2.86	2	0.36	201	7.88	.001
Positive feelings towards transfer	14.44	2	0.33	201	43.69	.000
Beliefs about transfer	15.73	2	0.22	201	73.03	.000
Ability to control transfer	7.34	2	0.59	201	12.41	.000
Transfer commitment	22.54	2	0.36	201	62.08	.000
Boss’s desire to transfer	27.67	2	0.73	198	37.69	.000
Clients’ desire to transfer	22.31	2	0.60	140	37.47	.000
Subordinates’ desire to transfer	4.40	2	0.44	61	9.99	.000
Pressure to transfer	1.70	2	0.26	201	6.50	.002
Intention to transfer	32.29	2	0.28	201	116.87	.000

*n* = 204, after screening

Table 5 provides common characteristics of these clusters. Although the factor’s values did not follow an increasing tendency in the clusters, each cluster had specific shared characteristics of the observed factors.

**Table 5:** Centroids of Final Clusters

Factor	Clusters		
	LowPT	MedPT	HighPT
Overcoming indeterminate obstacles during transfer	2.90	3.40	3.81
Decision-making in the transfer process	2.90	2.48	4.29
Negative feelings towards transfer	1.63	1.47	1.34
Overcoming work environment obstacles during transfer	2.66	2.63	3.28
Habits in the transfer process	3.56	3.87	3.92
Positive feelings towards transfer	3.53	4.14	4.41
Beliefs about transfer	3.37	4.09	4.23
Ability to control transfer	3.71	3.73	4.34
Transfer commitment	2.72	3.36	3.86
Boss’s desire to transfer	2.67	3.32	3.96
Clients’ desire to transfer	2.30	3.10	3.69
Subordinates’ desire to transfer	2.69	3.46	3.42
Pressure to transfer	1.55	1.76	1.42
Intention to transfer	3.19	4.09	4.50
Number of samples allocated to each cluster	25 (27%)	94 (46%)	55 (27%)

*n* = 204, after screening

LowPT = Low Predisposition to Transfer, which represents Cluster 1; MedPT = Medium Predisposition to Transfer, which represents Cluster 2; and HighPT = High Predisposition to Transfer, which represents Cluster 3

Cluster 1 included Low Predisposition to Transfer participants (LowPT) (27%); cluster 2 included Medium Predisposition to Transfer participants (MedPT) (46%), and cluster 3 included High Predisposition to Transfer participants (HighPT) (27%). We identified the cluster for each participant. Post-hoc analysis followed.

### Clusters’ Post-hoc Analyses

Contingency tables and inferential tests are presented in this section. Transfer was divided into thirds: low (≤ 2.33), medium (2.34—3.00) and high (> 3.00).

**Fig. 2:**
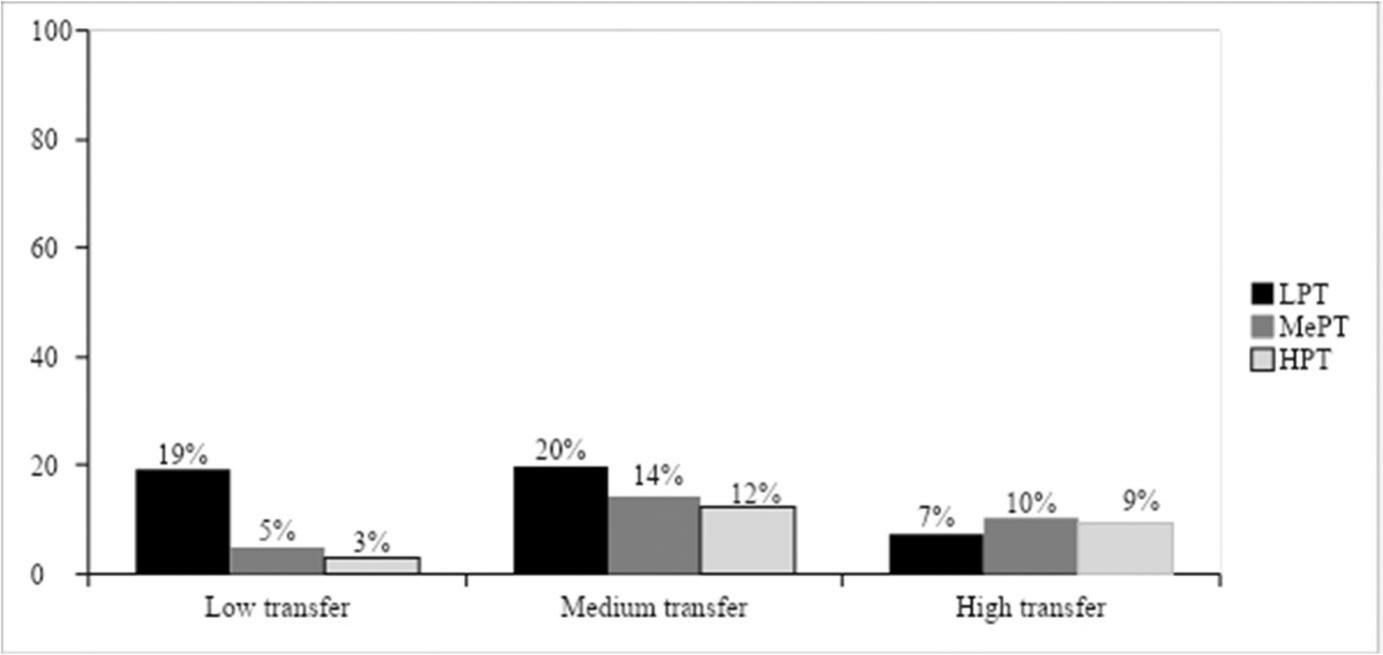
Percentage of Employees Classified by Transfer and Predisposition to Transfer. Note*.* *n* = 204, after screening. LPT = Low Predisposition to Transfer; MPT = Medium Predisposition to Transfer; and HPT = High Predisposition to Transfer

After applying the chi-square test, we found a significant association between the type of cluster belonging to ITI factors (*k* = 3) and the employees’ transfer level, being *X*^2^ (4) = 22.27, *p* < .001 (see Table 6 and Fig. 2).

**Table 6:** Contingency Table Between Transfer and Clusters

Transfer		Clusters			
		LowPT	MedPT	HighPT	Total
Low transfer	Count	39	10	6	55
	Expected count	25	16	14	55
	% within transfer levels	71	18	11	100
	% within clusters	42	17	12	27
	% of total	19	5	3	27
	Standardised residuals	2.7	-1.5	-2.0	
Medium transfer	Count	40	29	25	94
	Expected count	43	28	23	94
	% within transfer levels	43	31	27	100
	% within clusters	43	48	50	46
	% of total	20	14	12	46
	Standardised residuals	-0.5	0.3	0.4	
High transfer	Count	15	21	19	55
	Expected count	25	16	14	55
	% within transfer levels	27	38	35	100
	% within clusters	16	35	38	27
	% of total	7	10	9	27
	Standardised residuals	-2.1	1.2	1.5	
Total	Count	94	60	50	204
	Expected count	94	60	50	204
	% within transfer levels	46	29	25	100
	% within clusters	100	100	100	100
	% of total	46	29	25	100
	Standardised residuals	15	21	19	55

*n* = 204, after screening

*LowPT* Low Predisposition to Transfer; *MedPT* Medium Predisposition to Transfer; and *HighPT* High Predisposition to Transfer

Figure 2 shows the percentage of employees classified by transfer and predisposition to transfer, indicating that employees in cluster 1 were more related to low levels of transfer compared to the other two clusters in which employees expressed medium and high transfer levels. The motivational and emotional dynamics corresponding to low transfer were clearly defined by employees in cluster 1, with a LowPT. However, identifying a specific cluster showing common characteristics in their motivational and emotional dynamics associated with medium and/or high transfer was of increased difficulty.

Inferential test with the 14 ITI factors as dependent variables and the three types of clusters as a between-subjects variable was performed. One-way ANOVA explored the differences between the 14 ITI factors among the three clusters.

Results in Table 7 show that participants with LowPT had different means in 11 of 14 factors, compared to employees with MedPT. In addition, it shows MedPT and HighPT factor means, indicating differences in 11 factors. Finally, factor means for participants with LowPT and HighPT showed that 13 factors had different means between clusters.

**Table 7:** Differences Between the Factors of The Clusters

Factor	Mean of each Cluster (*p* value)		
	LowPT—MedPT	MedPT—HighPT	LowPT—HighPT
Overcoming indeterminate obstacles during transfer	2.90—3.40 (.000*)	3.40—3.81 (.000*)	2.90—3.81 (.000*)
Decision-making in the transfer process	2.90—2.48 (.004*)	2.48—4.29 (.000*)	2.90—4.29 (.000*)
Negative feelings towards transfer	1.63—1.47 (.174)	1.47—1.34 (.505)	1.63—1.34 (.003*)
Overcoming work environment obstacles during transfer	2.66—2.63 (1.000)	2.63—3.28 (.000*)	2.66—3.28 (.000*)
Habits in the transfer process	3.56—3.87 (.006*)	3.87 -3.92 (1.000)	3.56—3.92 (.002*)
Positive feelings towards transfer	3.53—4.14 (.000*)	4.14—4.41 (.045*)	3.53—4.41 (.000*)
Beliefs about transfer	3.37—4.09 (.000*)	4.09—4.23 (.410)	3.37—4.23 (.000*)
Ability to control transfer	3.71—3.73 (1.000)	3.73—4.34 (.000*)	3.71—4.34 (.000*)
Transfer commitment	2.72—3.36 (.000*)	3.36—3.86 (.000*)	2.72—3.86 (.000*)
Boss’s desire to transfer	2.67—3.32 (.000*)	3.32—3.96 (.001*)	2.67—3.96 (.000*)
Clients’ desire to transfer	2.30—3.10 (.000*)	3.10—3.69 (.005*)	2.30—3.69 (.000*)
Subordinates’ desire to transfer	2.69—3.46 (.001*)	3.46—3.42 (.000*)	2.69—3.42 (.000*)
Pressure to transfer	1.55—1.76 (.034*)	1.76—1.42 (.002*)	1.55—1.42 (.465)
Intention to transfer	3.19—4.09 (.000*)	4.09—4.50 (.000*)	3.19—4.50 (.000*)

*n* = 204, after screening

* *p* = .05. *LowPT*  Low Predisposition to Transfer; *MedPT* Medium Predisposition to Transfer; and *HighPT* High Predisposition to Transfer

**Fig. 3:**
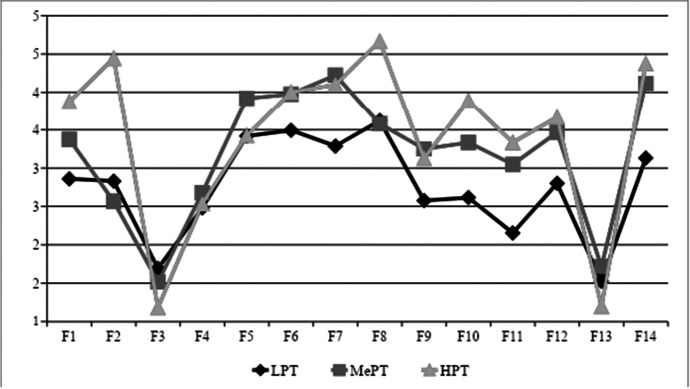
Low Transfer Participants’ Profile of Factors According to Their Predisposition to Transfer. *Note.* *n* = 204, after screening. F1: Overcoming indeterminate obstacles during transfer; F2: Decision-making in the transfer process; F3: Negative feelings towards transfer; F4: Overcoming work environment obstacles during transfer; F5: Habits in the transfer process; F6: Positive feelings towards transfer; F7: Beliefs about transfer; F8: Ability to control transfer; F9: Transfer commitment; F10: Boss’s desire to transfer; F11: Clients’ desire to transfer; F12: Subordinates’ desire to transfer; F13: Pressure to transfer; F14: Intention to transfer. LPT = Low Predisposition to Transfer; MePT = Medium Predisposition to Transfer; and HPT = High Predisposition to Transfer

Tables showed a greater difference in the factor profile between participants with LowPT and HighPT. In addition, low transfer showed the greatest differences among the levels of participants’ predisposition to transfer. Figure 3 shows that participants with LowPT might transfer in a low (19%) or medium level (20%) and suggests that participants showing low transfer are mostly employees with LowPT (19%) compared to MedPT (5%) or HighPT (3%). Figure 3 provides the participants’ common motivational factors that showed low transfer, according to their cluster (predisposition) in their factor scores.

Employees in the LowPT and HighPT clusters showed fewer stable scores on their motivational factors, noting that not all employees with low transfer had the same predisposition to transfer or that their motivational state was diverse.

## Discussion

We analysed the initial transfer intention factors and transfer following online training in participants from Spanish organisations by (1) categorising participants, through cluster analysis, according to their transfer level; and (2) by analysing the relation between the initial transfer intention factors and the different transfer levels.

Through cluster analysis we identified three groups of participants based on their initial predisposition to transfer (*k* = 3). This allowed us to identify in each of the clusters: low (LowPT), medium (MedPT) and high (HighPT) predisposition to transfer. Post-hoc analyses showed that differences were greater between employees with LowPT and HighPT, showing differences in all factors except for *pressure to transfer*. Participants with low transfer differed more in their intention to transfer than those with medium–high transfer.

Comparisons between low transfer employees’ profile of factors according to their cluster could help identify generalised trends and discover which factors should be subject to interventions to increase the effectiveness of the training.

The boss's desire to transfer was higher in the third cluster, however, that was not the case for perceived pressure to transfer. Some variables, such as job satisfaction, could be playing a role in these results, and therefore contribute to explain the different profiles in the clusters. Future research should explore the dynamics of these variables in their relation to the different transfer levels.

Results suggested motivational factors appear to influence transfer. In addition, the analysis identified key factors to enhance learning transfer in those employees who are expected to transfer less.

**Fig. 4:**
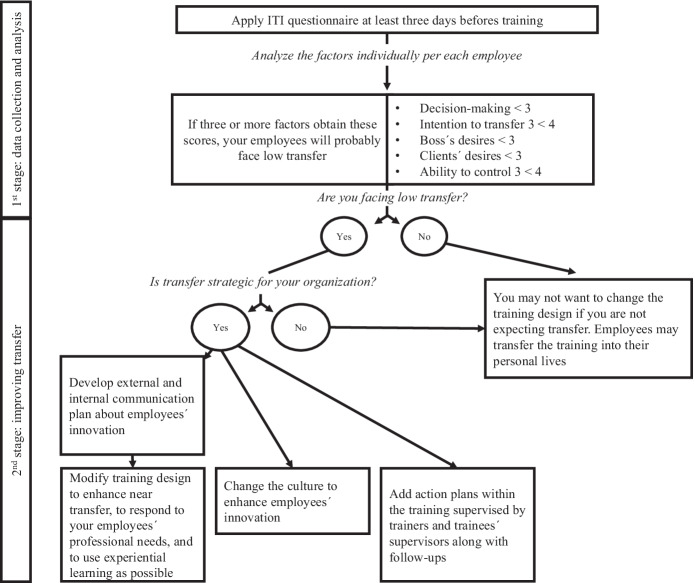
Strategies for LowPT Participants

After comparing LowPT and HighPT, all factors except pressure to transfer helped to differentiate between clusters. Examining participants’ factor-profile before training can identify meaningful information for training managers. Based on these results, a tree-map guide was developed (Fig. 4) to help practitioners diagnose potential low transfer before training, and to boost transfer.

If the training is not aligned with organisational strategies, practitioners also use training as a reward or to promote employees’ fulfilment. For instance, employees may participate in the activity due to their personal goals (Bosset & Bourgeois, 2015) and then training design may not need to change. However, when the training activity is aligned with organisational strategies, HRD agents can intervene through four strategies. First, they can develop external and internal communication plans about what employees have innovated in their workplace; the idea is to enhance the organisation’s transparency (Rawlins, 2008) and to acknowledge employees’ innovations, which can be useful as organisations’ public relations (Baker, 2008) and accountability (Gilpin et al., 2010). Second, they can design training content using examples that can be easily related to employees’ own workplace situation (Yamnill & McLean, 2001); design the training to ensure that it responds to employees’ professional needs (Brown, 2002); and use meaningful learning and instruction mechanisms such as experiential learning (Valkanos & Fragoulis, 2007), practice variability (Holladay & Quiñones, 2003) or error-encouragement framing (Bell & Kozlowski, 2008). Third, they can promote a culture change towards appreciating the value of innovation. The last strategy suggests adding action plans as part of the training design, supervised by trainers and trainees’ supervisors; these action plans are transfer implementation intentions (Friedman & Ronen, 2015). Furthermore, action plans must be accompanied by follow-up sessions (Richman‐Hirsch, 2001) to ensure its correct implementation. Through the implementation of the tree-map model, organisations can intervene before and during the training and transfer processes, increasing the chances of positive transfer of learning.

Advancing to solve the *transfer problem* (Saks & Burke, 2012) will decrease organisations’ concerns regarding their return on investment (Williams & Nafukho, 2015). Indeed, organisations cannot lose their money (Curry & Caplan, 1996) nor their resources in training activities that will not benefit them. Therefore “drawing attention to the importance of incorporating training initiatives at the highest level of strategic decision-making” is the first phase of our tree-map process model (Hurt, 2016, p.56). Findings from this study should help HRD units to make specific recommendations to their employees or division. One way to increase an organisation’s potential to achieve a better position in the labour market is to improve transfer of learning through well-planned and managed training (Kim & Ployhart, 2014).

This study answers the call for research in transfer evaluation through a trainee/centred focus (Massenberg et al., 2017; Poell, 2017), and it includes the study of the initial intention to transfer, which had also been claimed (Cheng et al., 2015; Testers et al., 2019). In addition, it takes two measures on each participant, answering the claim for longitudinal designs (Schoeb et al., 2020).

Specifically, this study carries out research in transfer in Southern Europe (Spain), making data from other regions than the USA and Western Europe public (Garavan et al., 2016). The study of transfer in Spain offers empirical data using participants whose first language is Spanish, complementing the pool of transfer evaluation data. These results are valid to the specific context of eLearning in Spain. Further research should investigate whether comparable results are obtained in different countries and cultures. Being Spain linked to most of Central and South America through the language, these results might also apply in those countries. Further research should explore whether these results could be generalised to the Spanish-speaking community, or are contingent to the context of Spain.

The study also contributes to the understanding of training transfer following eLearning interventions. Results can be used to better understand the eLearning modality and the controversy on training effectiveness based on the training modality (DeRouin et al., 2005; Lahti et al., 2014; Lee & Lin, 2013: Sitzmann et al., 2006). Further research should explore whether these results apply to face-to-face interventions.

We identified some limitations. First, using self-reports as the only technique to measure transfer. This is a common practice when objective measures involve high costs for (Chiaburu et al., 2010). To overcome possible bias, self-reports could be administered together with other techniques, such as peer evaluations, performance evaluation, or indicators of economic performance at an organisational level (De Grip & Sauermann, 2013; Segers et al., 2003). Second, it seemed easier to detect participants who will show low transfer levels than those who show medium or high transfer. This situation has also been found when other methodologies were applied, such as behaviour pattern analysis (e. g., Musso et al., 2013); indeed, predictive systems obtained clearer results when identifying lower performance because only more complex solutions were able to do so with equal precision for higher performance. Thus, this study showed that knowing which transfer predisposition profile a trainee will show at the beginning of a training activity can help practitioners to easily identify those that will have low transfer. In this manner, minimum conditions to transfer can be established. Third, the MTT (Quesada-Pallarès & Gegenfurtner, 2015) was not fully applied. We did not use the post-decisional stage, therefore, future research applying the entire model through a broader longitudinal design is needed. Fourth, while the sample was adequate, participants belonged to three organisations only, therefore, results do not represent all Spanish employees. More research in this topic is needed, including a larger number of organisations from different sectors to generalise the results to the Spanish context. Last, although performing a multilevel analysis could have been an interesting alternative due to the nature of data (22 online courses offered by three organisations and classified in three categories), the sample size did not allow us to perform these kinds of analyses (Maas and Hox, 2005). In addition, the proportion of the courses was disproportionate (hard skills: 53%; technological skills: 31%; soft skills: 16%). Future research could include a larger sample that allows multilevel analyses on a sufficient sample.

To conclude, there are two options to design further steps for this research. First, additional post-hoc analyses could be applied to this data, such as discriminant analysis or regression models among employees with low transfer level and the three profiles detected. Secondly, more advanced techniques could be used, like predictive systems using neural networks. This latter technique would allow us to obtain a mathematical model with predictive value, which could handle the vast array of potential predictors and their complex interactions. Second, we could perform further studies to confirm these behaviour trends and to explore the typological groups of each cluster (personality traits, job motivations, educational profile, etc.) and to add other relevant aspects, such as the learning path, that may help us understand trainees’ learning-path strategy (Poell, 2017) using a within-person centred approach (Huang et al., 2017). Also, we could conduct a study using only the significant factors from the MTT (Quesada-Pallarès & Gegenfurtner, 2015) that help us differentiate among clusters, so data is simpler. To do so though, a new validation of the reduced model should be conducted first. Furthermore, cross-cultural, and representative studies that would allow us to generalise these results or to explore other profiles are needed.
